# Feasibility of Droplet Digital PCR Analysis of Plasma Cell-Free DNA From Kidney Transplant Patients

**DOI:** 10.3389/fmed.2021.748668

**Published:** 2021-10-08

**Authors:** Barbara Jerič Kokelj, Maja Štalekar, Sebastian Vencken, David Dobnik, Polona Kogovšek, Matjaž Stanonik, Miha Arnol, Maja Ravnikar

**Affiliations:** ^1^Department of Biotechnology and Systems Biology, National Institute of Biology, Ljubljana, Slovenia; ^2^GenePlanet, Ljubljana, Slovenia; ^3^Department of Nephrology, University Medical Centre Ljubljana, Ljubljana, Slovenia; ^4^Faculty of Medicine, University of Ljubljana, Ljubljana, Slovenia

**Keywords:** kidney transplantation, droplet digital PCR, plasma cell-free DNA, minor allele quantification, assay evaluation, graft health monitoring

## Abstract

Increasing research demonstrates the potential of donor-derived cell-free DNA (dd-cfDNA) as a biomarker for monitoring the health of various solid organ transplants. Several methods have been proposed for cfDNA analysis, including real-time PCR, digital PCR, and next generation sequencing-based approaches. We sought to revise the droplet digital PCR (ddPCR)-based approach to quantify relative dd-cfDNA in plasma from kidney transplant (KTx) patients using a novel pilot set of assays targeting single nucleotide polymorphisms that have a very high potential to distinguish cfDNA from two individuals. The assays are capable of accurate quantification of down to 0.1% minor allele content when analyzing 165 ng of human DNA. We found no significant differences in the yield of extracted cfDNA using the three different commercial kits tested. More cfDNA was extracted from the plasma of KTx patients than from healthy volunteers, especially early after transplantation. The median level of donor-derived minor alleles in KTx samples was 0.35%. We found that ddPCR using the evaluated assays within specific range is suitable for analysis of KTx patients' plasma but recommend prior genotyping of donor DNA and performing reliable preamplification of cfDNA.

## Introduction

Cell-free DNA (cfDNA) is gaining more and more attention and research toward its use as a diagnostic and prognostic biomarker for various medical conditions—prenatal diagnostics, oncology and solid organ transplantation [reviewed in (1–3)]. Fragmented DNA enters the bloodstream and other body fluids and provides genomic and epigenomic information of the source tissue through minimally invasive liquid biopsy.

While renal biopsy is still the gold standard for monitoring and confirming transplanted kidney (KTx) health, other non-invasive screening tests are being sought that would replace it or reduce its need. Unless the graft was donated by an identical twin, its genotype differs from that of the patient. Taking advantage of the different genotypes, donor-derived cfDNA (dd-cfDNA) secreted by the graft can be distinguished from the background of recipient-derived cfDNA secreted by other tissues. Analysis of dd-cfDNA has been shown to have the potential to detect cases of rejection episodes, with patients with rejection having higher plasma dd-cfDNA levels than patients with stable grafts without rejection (4–7). A schedule of longitudinal dd-cfDNA analyses to monitor graft health was proposed, along with additional indicated analyses when graft damage is suspected and after changes in immunosuppression (8).

Donor-derived cfDNA can be quantified by several methods, including real-time PCR (qPCR) (9, 10), digital PCR (11–14) and next generation sequencing (4, 5, 7). Single nucleotide polymorphisms (SNPs) have been most commonly studied to distinguish recipient and donor DNA, but other polymorphisms—human leukocyte antigen mismatch (13), indels (9, 14) and copy number variations (15, 16)—have also been investigated. In this report, we used droplet digital PCR (ddPCR) as a method for quantifying dd-cfDNA in KTx based on SNP differences between donor and patient derived DNA with a critical proof-of-principle analysis of donated plasma samples using a novel set of SNP assays and also address the need for assay evaluation. In digital PCR, the reaction mixture is divided into individual PCR reactions (droplets in the case of ddPCR), which is expected to result in one or no template DNA molecules per reaction, and the Poisson distribution is adopted for absolute quantification of the template [reviewed in (17)]. Digital PCR has the advantage over qPCR of being more accurate at low target concentrations and not using standard curves, and over next generation sequencing of being less expensive, having a shorter turnaround time, and being easier to interpret without implementing bioinformatics algorithms.

## Materials and Methods

### SNP Selection

One thousand Genomes Project VCF files for all chromosomes were downloaded^1^ (18). Data were filtered stepwise for biallelic SNPs, European minor allele frequency > 0.40, global minor allele frequency > 0.45 and global fixation index < 0.02. Distances (CA01dist, CA01score) from SNPs to the nearest cfDNA fragment peaks were calculated using the CA01.bb file (18) and data were filtered for CA01dist < 19 and CA01score > 200. Using USCS Table Browser, all SNPs overlapping with repetitive elements were identified and removed. The surrounding sequences of the remaining 1,051 SNPs were screened for potentially useful primer pairs and probes using Beacon Designer 8.4 and SNPs for which no amplicons were found were removed. SNPs with overlap of potential amplicons with off-target SNPs in potential primer or probe sequences were identified using UCSC Table Browser and discarded. Screening for off-target potential of predicted primers was performed using Primer-BLAST. IDT OligoAnalyzer 3.1 was used to calculate ΔG of primer dimers, hairpin Tm and secondary structures. Based on these characteristics of the identified potential assays, SNPs were ranked and 43 with the best potential were selected.

### gBlocks

One hundred sixty-one blood pressure dsDNA synthetic gBlock Gene Fragments (Integrated DNA Technologies) mimicking natural short DNA fragments were designed for each allele of the selected SNPs. Each gBlock had the same sequence as the corresponding genomic region and contained the SNP assay amplicon approximately in the middle with at least 29 bp of flanking sequence on each side. Prior to use, gBlocks were diluted in 2 mg/ml Sheared Salmon sperm DNA (Invitrogen).

### Collection and Processing of Blood Samples

Clinical samples were collected and processed in accordance with the approval of Slovenian National Medical Ethics Committee (No. 0120-479/2016-2, KME 42/09/16). Approximately 10 ml of blood was collected in Cell-Free DNA BCT tubes (Streck) and stored at room temperature until further processing (maximum 72 h after blood draw). Plasma was separated from blood by two consecutive centrifugation steps (10 min at 1,600 g and 10 min at 16,000 g) at room temperature. The supernatant was immediately transferred to a fresh tube to reduce contamination of the plasma with cells and stored at −80°C until cfDNA extraction. The buffy coat layer was harvested and stored at −20°C until gDNA extraction.

### DNA Extraction

Genomic DNA (gDNA) was isolated from a maximum of 200 μl of buffy coat using QIAamp DNA Mini Kit (Qiagen) according to the manufacturer's instructions. gDNA was eluted in 200 μl of the supplied AVE elution buffer. The isolated gDNA was stored at −20°C until further use.

In our kit comparison study, cfDNA was extracted from three 1 ml aliquots of the same plasma sample from apparently healthy donors (*N* = 10) according to the manufacturer's instructions using each kit: QIAamp Circulating Nucleic Acid Kit together with QIAvac 24 Plus Vacuum System (CNA; Qiagen), QIAamp MinElute ccfDNA Kit (ME; Qiagen) and MagMAX Cell-Free DNA Isolation Kit (MM; Thermo Fisher Scientific). Extractions with different kits were performed on three separate days, with one kit per day comprising all test samples. To ensure repeatability between laboratories, extractions were repeated in a different laboratory with a smaller subset of samples.

For isolation of cfDNA from 4 ml of plasma from KTx patients, the CNA kit was used according to the manufacturer's instructions. cfDNA was eluted in 45 μl of AVE buffer, aliquoted and stored at −20°C until further analysis.

The concentration of extracted DNA was determined fluorometrically using Qubit dsDNA HS Assay Kit and Qubit 3 Fluorometer (Thermo Fisher Scientific).

Capillary electrophoresis was performed using HT DNA High Sensitivity LabChip Kit and Caliper LabChip GX instrument and analyzed using LabChip GX Software Version 5.2.2009.0 (all from Perkin Elmer).

### qPCR

The qPCR reactions were performed in a 384-well format on the ViiA 7 or QuantStudio 7 Flex Real-Time PCR System using software-implemented experimental settings for genotyping. Ten microlitre qPCR reactions consisted of TaqMan Genotyping Master Mix (Themo Fisher Scientific), TaqMan Custom SNP Genotyping Assay (Themo Fisher Scientific; Supplementary Table 1), 5 ng of gDNA and nuclease-free water to final volume. The thermal profile included pre-read phase (60°C, 30 s), hold phase for denaturation (95°C, 10 min), 40 cycles of PCR phase (95°C, 15 s; 60°C, 1 min), followed by post-read phase (60°C, 30 s); the ramp rate was 1.6°C/s. Genotypes were called automatically based on the analysis algorithm embedded in the software using real-time dRn data and a quality score of 95.0.

### ddPCR

The QX200 AutoDG ddPCR System (Bio Rad) was used for droplet generation and readout. Reactions in the final volume of 20 μl consisted of ddPCR Supermix for Probes (No dUTP, Bio-Rad), TaqMan Custom SNP Genotyping Assay (Themo Fisher Scientific, Supplementary Table 1), DNA template and nuclease-free water to the final volume. In the case of cfDNA analysis, 9 ul of cfDNA was used per reaction. Droplets were generated using the Automated Droplet Generator (Bio-Rad). The following cycling conditions were used on the C1000 or T100 Thermal Cycler (Bio-Rad): Initial denaturation at 95°C for 10 min was followed by 40 cycles of 94°C for 30 s, 60°C for 60 s and 1 cycle of enzyme deactivation at 98°C for 10 min. The ramp rate was 1.5°C/s. After cycling, plate was transferred to QX200 Droplet Reader (Bio-Rad).

Positive droplets were distinguished from negative droplets by applying a threshold for fluorescence amplitude in QuantaSoft software (Bio-Rad), version 1.7.4. The threshold value was set manually. The same threshold was used for each channel/assay throughout the reaction plate and similar thresholds were enforced for the same channel/assay in different reaction plates. Reactions with <10,000 detected droplets and saturated reactions with more than 100,000 target copies were rejected and excluded from the analysis. Data were exported and allele fractions along with coefficient of variation (CV) were calculated using Microsoft Excel software. A droplet volume of 0.739 nl was considered for the calculation of absolute measured copy number (19).

### PCR Preamplification of cfDNA

Targeted multiplex PCR preamplification was prepared using Q5 Hot Start High-Fidelity Master Mix (New England Biolabs), 500 nm final concentration of each of the primers (Integrated DNA Technologies) used in assays targeting rs1707473, rs7687645, rs1420530 and rs6070149 (Supplementary Table 1), DNA template and nuclease-free water. The following reaction conditions were used in the C1000 or T100 Thermal cycler (Bio-Rad): an initial denaturation at 98°C for 30 s was followed by 8 cycles of 98°C for 10 s, 60°C for 30 s and 72°C for 20 s, and a final extension at 72°C for 2 min. The PCR products were then stored at −20°C until ddPCR analysis. Prior to ddPCR analysis, PCR products were diluted 10-fold with nuclease-free water.

### Statistical Analysis

All tests were performed in RStudio, version 1.4.1106, using the packages ggpubr (version 0.4.0) and rstatix (version 0.7.0). The normality of the data was interrogated using the Shapiro–Wilk test. To compare the extraction yields of the three kits, the average yields across the three replicates of each sample were calculated and any influence of the kit on the extraction yield was investigated using the one-way repeated measures ANOVA test. Yields of KTx samples were compared to yields of samples from healthy donors using the Mann Whitney U test. For multiple comparisons, the Benjamini-Hochberg adjustment of *P*-values was used.

## Results

### SNP Selection and Performance of SNP Detection Assays

Our approach to selecting biallelic SNP candidates that could more readily distinguish the DNA of one individual from another included a wide range of criteria. Considering high minor allele frequency, low global fixation index, proximity to cfDNA fragment peaks, no overlap with repetitive elements and comprehensive *in silico* amplicon analysis, 43 promising biallelic SNPs are suggested in Table 1. None of the SNPs have known clinical significance and they are all positioned in non-coding regions, either outside any gene regions or in introns or untranslated regions.

**Table 1:** Proposed biallelic SNPs for distinguishing the cfDNA of two individuals.

**SNP ID**	**Alleles**	**Chr: position (GRCh38.p12)**	**European MAF**	**Global MAF**	**Position in relation to genes**
rs1935037	A, T	1:223323387	T = 0.4602	T = 0.4942	SUSD4 intron
rs10168994	T, C	2:193486802	C = 0.4026	C = 0.4754	/
rs745581	C, T	2:2729334	T = 0.4881	T = 0.4984	/
rs6546954	C, G	2:75182348	C = 0.4702	G = 0.4585	TACR1 and LOC105374811 intron
rs1707473*	T, G	3:146133737	T = 0.4950	G = 0.4930	PLOD2 intron
rs6775401	A, G	3:183462906	G = 0.4682	G = 0.4718	/
rs7431017*	A, C	3:76502252	A = 0.4811	C = 0.4559	ROBO2 intron
rs9289628*	C, T	3:98548445	T = 0.4901	C = 0.4896	/
rs7687645*	T, C	4:70350352	T = 0.4523	C = 0.4970	/
rs615954	G, C	5:123144242	C = 0.4761	C = 0.4884	PRDM6 intron
rs10078699	C, T	5:155125342	T = 0.4940	T = 0.4507	/
rs153749	A, G	5:171754251	G = 0.4205	G = 0.4764	/
rs2561182	G, C	5:69437818	C = 0.4911	C = 0.4784	MARVELD2 intron
rs7746234	C, T	6:138498373	C = 0.4066	T = 0.4930	NHSL1 intron
rs2517471	G, C	6:31084321	G = 0.4254	C = 0.4952	/
rs9656097	T, C	7:104526571	C = 0.4076	C = 0.4708	LHFPL3 intron
rs751966	G, A	7:135879129	A = 0.4871	A = 0.4679	/
rs2691527*	C, T	7:78173386	C = 0.4881	T = 0.4647	MAGI2 intron
rs6557883	T, C	8:26127967	C = 0.4811	C = 0.4992	LOC105379335 non-coding transcript
rs4733016	C, T	8:26991085	T = 0.4722	T = 0.4806	/
rs2974298	C, T	8:42518957	T = 0.5000	T = 0.4850	SLC20A2 intron
rs7011817	G, C	8:60212109	G = 0.4771	C = 0.4667	CA8 intron
rs2246293	C, G	9:104928557	G = 0.4871	G = 0.4892	LOC105376196 intron; ABCA1 upstream transcript
rs10901532	T, C	10:126136495	C = 0.4414	C = 0.4591	ADAM12 intron
rs7117897	T, C	11:129853970	C = 0.4632	C = 0.4561	TMEM45B intron
rs1166235	G, A	12:126874295	A = 0.4563	A = 0.4888	LINC02372 intron; LOC105370061 upstream transcript
rs10843022	C, G	12:27904982	G = 0.4414	G = 0.4503	/
rs7972248	C, T	12:3515931	C = 0.4980	T = 0.4579	PRMT8 intron
rs4770602	C, G	13:24136006	G = 0.4901	G = 0.4617	SPATA13 intron
rs7321093	T, C	13:28573704	C = 0.4831	C = 0.4872	LOC105370135 upstream transcript
rs2799341	T, C	13:53072591	C = 0.4036	C = 0.4501	/
rs3784165	G, C	14:31088123	C = 0.4135	C = 0.4696	AP4S1 intron
rs8014321	T, C	14:91501322	C = 0.4751	C = 0.4916	PPP4R3A intron
rs10148348	T, C	14:92866186	T = 0.4732	C = 0.4950	/
rs1420530*	C, T	16:52524817	T = 0.4821	T = 0.4892	TOX3 intron
rs1392494	G, A	16:59678252	A = 0.4781	A = 0.4894	/
rs1384366	G, A	17:78733702	A = 0.4553	A = 0.4665	CYTH1 intron
rs4789798	C, G	17:82573767	G = 0.4254	G = 0.4603	FOXK2 intron
rs12605230	G, C	18:38219647	C = 0.4871	G = 0.4996	/
rs6075029	C, T	20:16200100	T = 0.4980	T = 0.4573	/
rs6050798	C, T	20:2641352	T = 0.4891	T = 0.4681	TMC2 3' UTR; LOC105372505 intron
rs6070149*	C, T	20:57554682	T = 0.4791	C = 0.4732	/
rs243593	G, A	21:17783905	A = 0.4831	A = 0.4802	C21orf91-OT1 intron

SNPs marked with asterisk (*) were selected for assay design and evaluation of minor allele quantification performance. The European and global minor allele frequencies (MAF) of the SNPs were derived from the 1,000 Genomes Project (18).

Of these, 7 SNP loci were randomly selected for assay design and evaluation of their performance. First, assays were tested for their performance characteristics in a genotyping qPCR. Allelic discrimination plots were examined as a checkpoint for reaction errors. Six of the assays tested showed good cluster separation with dense clusters at the expected positions in the allelic discrimination plots. No inhibition of qPCR reactions with extracted gDNA samples was detected. Cluster separation of the assay targeting rs7431017 was not as clear, but still sufficient.

Next, the assays were tested with ddPCR. Again, cluster separation was checked and the assay targeting rs7431017 was omitted due to cluster overlap. A representative scatterplot showing good separation of positive and negative droplets is shown in Supplementary Figure 1A. Interestingly, we noticed remarkable rain when analyzing gBlocks with all of our SNP assays, while there was significantly less rain present when analyzing gDNA or cfDNA.

To evaluate the performance of the ddPCR assay targeting rs1707473 in a scenario mimicking presence of donor-derived DNA in a background of recipient's DNA, 0.1–50% ratios of alleles G and T (0.1–33% minor allele fractions) were simulated with mixtures of synthetic gBlocks of the two alleles. The assay provides a linear signal over the whole wide tested range with *R*^2^ of 0.999 for the allele T in a background of the allele G and 0.998 for the allele G in a background of the allele T (Supplementary Figure 1B).

Using gBlocks, CV values were also determined at 1% simulated dd-cfDNA with three different low copy number mixtures: 5/500, 10/1,000 and 20/2,000 copies of allele 1/allele 2 per reaction. Each allele mixture was prepared and analyzed in 6 ddPCR reactions. CV was calculated for each of the alleles in the mixture and the results for assay targeting rs1707473 are summarized in Supplementary Table 2. As expected, the minor allele contributes the most to the overall CV value of the assay in duplex format, as the CV values are much lower for the major allele, which is present in higher copies per reaction (Supplementary Table 2). The limit of quantification of this assay, where CV falls below the set 20%, was found to be between 10 and 20 copies of the target per reaction. The limit of detection for the same assay was found at 5 copies per reaction (data not shown).

To assess possible bias due to synthetic gBlocks, the assay performance characteristics were also confirmed in a ddPCR reaction with cfDNA isolated from heterozygous healthy blood donors in the range of ~3–60 copies per 20 μl reaction. In this case, the limit of quantification was determined to be in the range of 15–32 copies per reaction (data not shown).

Quantification of very low abundance alleles by SNP analysis (analogous to detection and quantification of the mutant in a high background of a wild-type allele) requires additional assessment of assay performance. A high background of the major allele vs. progressively lower abundance of the minor allele presents a potential hazard: a false positive signal (FPS) in the minor allele detection channel. The FPS should be measured and the detection cut-off for the minor allele defined with respect to the FPS. Therefore, we continued the characterization of the rest of the assays using artificial mixtures of gDNA and gBlocks with a constant background of ~ 50,000 copies of major alleles (≈ 165 ng of human DNA) and a variable proportion of 0–0.5% copies of minor alleles. Analysis of such samples allowed us to be on the theoretically safe side for accurate quantification of 0.1% minor allele copies (50 copies per reaction) or even less. The samples to which no minor allele copies were added were used to estimate the FPS for each allele of all characterized assays.

The FPS is assay-dependent, reaching up to 0.14% in the case of assay targeting rs7687645, allele C (Table 2). To quantify the presence of minor alleles in the tested sample, we propose to subtract the FPS. In this way, the minor allele was successfully detected in all cases down to 0.1% minor allele copies without overlapping with the FPS. The data suggest reliable quantification of minor allele frequency down to 0.1–0.5%, depending on the assay/allele (CV ≤ 20%).

**Table 2:** Characteristics of SNP ddPCR assays defined by measuring copies of alleles in mixtures of gDNA/gBlocks with a background of 50,000 copies of major alleles per ddPCR reaction.

**Assay target**	**Target allele**	**FPS [%]**	**LOD [%]**	**LOQ [%]**
rs1707473	G	0.00	<0.09	<0.09
	T	0.02	<0.13	0.13–0.23
rs2691527	C	0.03	0.05–0.08	0.03–0.08
	T	0.04	0.05–0.08	0.27–0.42
rs7687645	C	0.14	0.08–0.1	0.21–0.47
	T	0.03	0.08–0.1	0.16–0.38
rs1420530	C	0.03	0.05–0.08	0.08–0.26
	T	0.03	0.05–0.08	0.05–0.09
rs9289628	C	0.09	0–0.05	0.13–0.22
	T	0.07	0.05–0.08	0.13–0.24
rs6070149	C	0.03	0.05–0.1	0.08–0.16
	T	0.03	0.025–0.05	0.24–0.5

*For each assay/allele, the false positive signal (FPS) of the background without target allele was measured. The limit of detection (LOD) was defined in a range of the minor allele proportion where the target signal significantly exceeds the false positive signal. The limit of quantification (LOQ) was defined in a range of minor allele proportion where the coefficient of variation of the ddPCR measurements did not exceed 20%. To narrow down or precisely define the ranges of LOD and LOQ, further measurements should be performed in the respective ranges of minor allele proportions*.

### Comparison of cfDNA Extraction From Plasma

**Figure 1:**
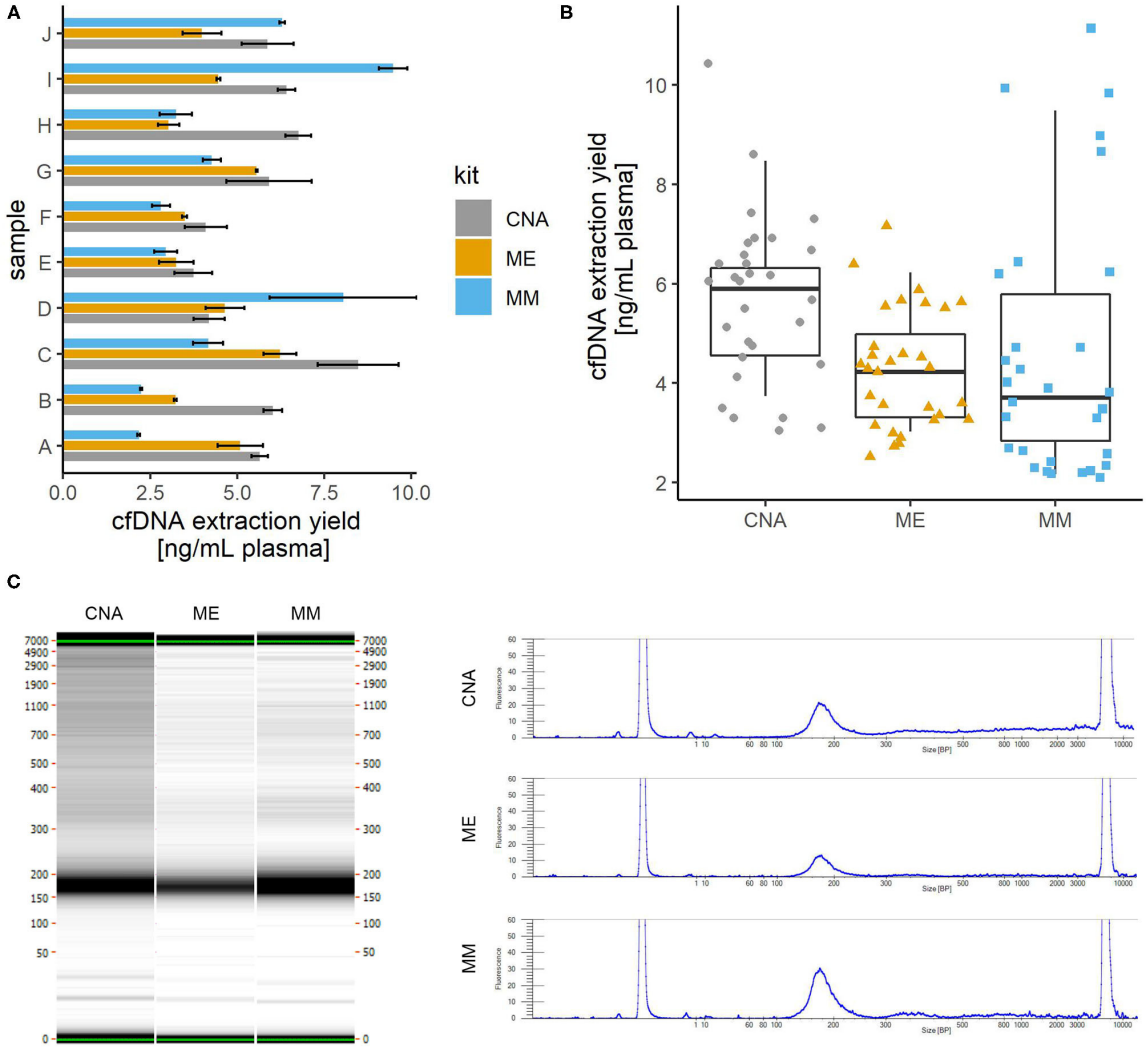
Comparison of cfDNA isolation from plasma using three different commercially available kits. cfDNA was isolated from 1 ml of plasma using each kit in three replicates. **(A)** The average yield for each plasma sample. Error bars show the standard deviation of three replicates. **(B)** Distribution of yield of cfDNA isolation with three kits. Colored shapes show the individual yields of all samples/replicates and boxplots show the average yields across replicates. No significant differences were observed between the yields obtained with the three kits tested. **(C)** Comparison of fragment sizes. The left panel shows the capillary electrophoresis gel for one of the three replicates of the same sample extracted with three different commercial kits. On the right, the electropherogram of the same gel shows that the fragment size profile is very comparable between the kits, with a major peak around 170 bp representing mainly the mononucleosomal cfDNA fraction and a minor peak around 340 bp (probably dinucleosomal cfDNA). CNA, QIAamp Circulating Nucleic Acid Kit; ME, QIAamp MinElute ccfDNA Kit; MM, MagMAX Cell-Free DNA Isolation Kit.

We compared the yields of cfDNA isolation from plasma using three widely used commercial kits. Blood was collected from 10 apparently healthy donors, plasma was divided into 1 ml aliquots, and cfDNA was isolated from 3 aliquots using each kit. The results are shown in Figures 1A,B. The cfDNA yield was comparable as no significant differences were found between kits (one-way repeated measures ANOVA; *p* = 0.152), but appeared to be best with the CNA kit with a mean of 5.7 ng/ml plasma and a median of 6.1 ng/ml, compared to ME with a mean and median of 4.3 ng/ml plasma and MM with a mean of 4.6 and a median of 3.7 ng/ml plasma. Intersample variability appeared to be highest with the MM kit. The fold difference between the highest and lowest yields obtained was 5.3 with MM, 3.4 with CNA and 2.8 with ME. Intrasample variability (reproducibility between aliquots) was comparable for all three kits (mean coefficient of variance: CNA 18%, ME 13%, and MM 15%). It is possible that more reproducible results could be obtained with an automated protocol available for all three kits but not tested in our laboratory.

Fragment size analysis also revealed no significant differences between the kits tested (Figure 1C). Due to the lack of detectable high molecular weight fragments, no further assay-based quantitative analysis was performed to assess genomic contamination.

Given the lack of significant differences in DNA yields measured by Qubit fluorometer between the kits tested and in agreement with the comparable results obtained by capillary electrophoresis, accurate yields were determined by absolute copy number quantification by ddPCR only for the cfDNA extracted by the CNA kit, which was selected for further use in our work. Very good correlation was found between yields measured by Qubit and DNA copy number measured by ddPCR using different SNP detection assays (data not shown). No significant inhibition of ddPCR reactions was observed when analyzing cfDNA samples isolated by column-based cfDNA extraction using the CNA kit.

### cfDNA From Plasma of Kidney Transplant Patients

**Figure 2:**
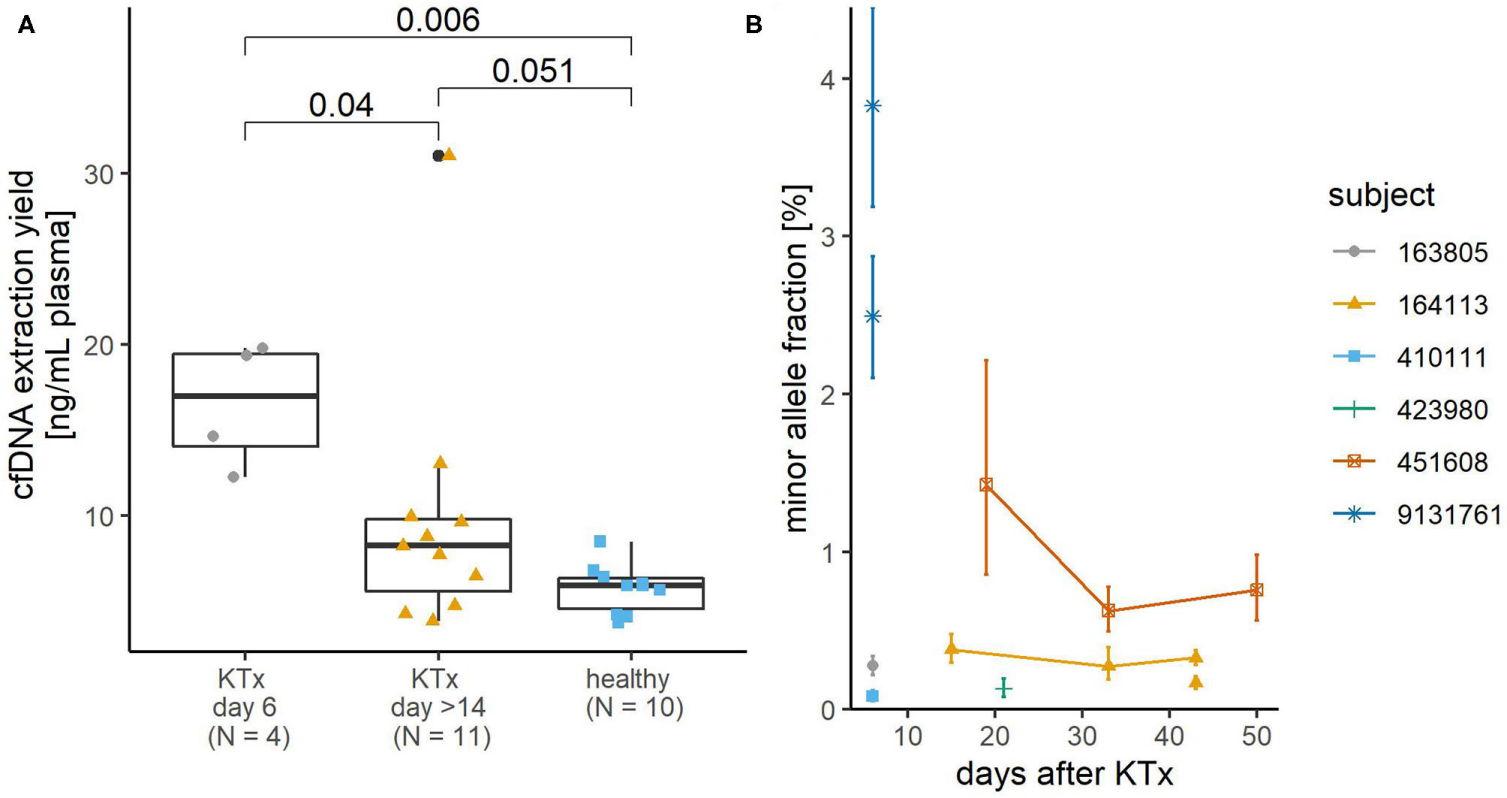
Yield of plasma cfDNA from kidney transplant (KTx) patients and its donor-derived minor allele content. **(A)** The KTx cfDNA yield was higher than that of healthy volunteers. Colored shapes show the yields of each sample and boxplots summarize their distribution. *P*-values of pairwise comparisons using Mann Whitney *U*-test and Benjamini-Hochberg adjustment are shown. **(B)** Minor allele content in plasma cfDNA of KTx patients. The detected minor alleles are from the donor, but the zygosity status of the donor DNA is not known, so the exact proportion of donor-derived DNA would be 2-fold in the case of heterozygous donor DNA. For two samples (subject ID 9131761, 6 days after KTx and subject ID 164113, 43 days after KTx), the minor allele proportion was determined using two SNP assays, hence two values for the minor allele proportion of each sample. Error bars represent Poisson confidence intervals. In three cases where the confidence intervals are wider (subject ID 9131761, 6 days after KTx and subject ID 451608, 19 days after KTx), minor allele proportions were determined in non-preamplified cfDNA samples. In the other cases, ddPCR analysis was performed after targeted multiplex PCR preamplification of cfDNA.

Next, cfDNA was extracted with the CNA kit from 15 4 ml plasma samples donated by 8 KTx patients. Four patients donated blood on day 6 after transplantation, and 4 patients donated 3 or 2 samples at different time points more than 14 days and within 13 months after transplantation, with at least 10 days between consecutive donations. Extraction yields ranged from 15.3 to 124.2 ng with a median of 38.4 ng and a mean of 46.3 ng, corresponding to ~ 4,600–37,600 haploid genome equivalents with a median of 11,600 and a mean of 14,000 haploid genome equivalents. On average, we extracted more cfDNA from plasma of KTx patients than from plasma of apparently healthy donors in the above test experiment (*p* = 0.007). The increase in cfDNA yield was particularly pronounced shortly after KTx (*p* = 0.006), whereas the mean extraction yield from samples collected more than 14 days after KTx was not statistically significantly different from the mean extraction yield from samples from healthy subjects (*p* = 0.51; Figure 2A).

### Minor Allele Proportions in KTx Samples

At least the genotype of the patient and preferably that of the donor should be known to allow quantification of the dd-cfDNA. To identify the patient's genotype, the gDNA extracted from buffy coat from KTx patients was genotyped by qPCR at all six SNP sites. At least one homozygous SNP was detected for each patient. No donor tissue was available for genotyping. The corresponding plasma cfDNA was analyzed by ddPCR with assays targeting SNPs for which the patient's DNA was homozygous to search for and quantify the presence of foreign donor-derived alleles. Donor-derived alleles could only be quantified in three samples with relatively high minor allele proportions−1.43% and higher. Most other samples were subsequently PCR-preamplified and reanalyzed by ddPCR. The quantifiable minor allele proportions in these samples ranged from 0.08 to 0.76% (Figure 2B).

## Discussion

We have selected and proposed a new biallelic SNP set that has a very good potential to distinguish the DNA of two individuals. To our knowledge, this is the first reported SNP set matched to cfDNA fragment peaks, indicating increased protection of DNA from digestion (20) and enabling the design of PCR SNP detection assays in regions of reduced fragmentation. As a proof of principle, we evaluated the performance of six SNP detection assays that are now ready for use. In a background of 50,000 major allele DNA copies, they are able to quantify at least 0.5% and some even 0.1% minor allele copies. The most commonly used set of SNP assays for this purpose is reportedly capable of detecting minor allele content down to 2% with a CV of <15% (11), but has nevertheless been used to quantify 0.1%.

For use in clinical practice, an assay should be able to accurately detect dd-cfDNA fractions above the established threshold that marks possible rejection and half the threshold to allow analysis of informative heterozygous donor SNPs. Thus, considering the most commonly proposed threshold of 1% for KTx (4, 5, 7), an assay should reliably quantify down to 0.5% minor allele content. However, some studies suggest an even lower threshold (6, 21–23). It has been suggested that plasma dd-cfDNA fraction could be measured by ddPCR analysis of SNPs without prior knowledge of donor genotype (11), but low cfDNA levels and low dd-cfDNA fractions in KTx and also heart transplant patients argue against this.

Quantification of low-copy DNA is limited not only by assay performance characteristics but also, and probably more so, by stochastic effects associated with low copy numbers and subsampling, which contribute significantly to measurement uncertainty at low limits of detection (24–27). Assuming 30 minor allele copies ( 100 pg of human DNA) as a theoretical lower limit for reliable quantification, a minor allele content of 0.1% requires ~100 ng of cfDNA, which is not trivial to obtain from plasma of KTx patients. Considering the yield of cfDNA that we extracted from 4 ml of plasma from KTx patients, the theoretical limit of quantification for corresponding samples is in the range of 0.08–0.65%. Thus, based on our observation, sample size (i.e., volume of blood collected and cfDNA content in blood) is the most important limiting factor in cfDNA analysis.

The cfDNA extraction yield from plasma of our group of KTx patients was similar to that previously reported for KTx patients (21) but lower than that reported for liver transplanted patients (28). We observed a higher cfDNA extraction yield from plasma of KTx patients compared to plasma cfDNA from apparently healthy individuals, which is in agreement with Schütz et al. who also reported a higher total cfDNA in a group of KTx patients compared to a healthy control group or a group with other medical conditions (29).

Half of the KTx plasma cfDNA samples included in our study had a minor allele content of <0.35%, which is consistent with the reported median baseline values of 0.21–0.46% in KTx [reviewed in (8)]. The calculated inherent theoretical limit of minor allele quantification was higher than this in 4 of 15 (27%) cfDNA samples, implying that in some cfDNA samples the actual minor allele content is lower than its theoretically derived quantifiable limit. In such samples, reliable determination of the donor genotype would be hampered because it depends on accurate quantification of the minor allele content. Although the results of such samples would likely be adequately classified as sub-threshold (non-rejection), subsequent samples from the same patient would need to be repeatedly analyzed with many SNP assays until the donor's cfDNA content is high enough to allow reliable determination of his genotype. On the other hand, prior knowledge of the genotype would allow analysis with fewer assays and reduce overall costs. Additional one-time genotyping would also not significantly increase the turnaround time of the analysis. Therefore, in the case of KTx and possibly other solid organ transplants with low expected levels of donor DNA, such as heart transplants, other sources of donor DNA should preferably be genotyped in advance. Urine could be a suitable and readily available source of transplanted kidney DNA as suggested by Oellerich et al. (6).

Our results suggest that plasma cfDNA from KTx patients should first be preamplified in order to quantify minor allele content, which agrees well with a study in which donor-derived DNA was almost undetectable in non-preamplified plasma cfDNA from KTx patients (12). If only detection of a minor allele fraction above the 1% threshold was required to identify rejection cases, the yield of cfDNA extraction was high enough to allow analysis of ~10 ng in a ddPCR reaction, and the donor genotype was known, preamplification could theoretically be omitted. Otherwise, an evaluated reliable preamplification should be performed that should not cause a significant shift in allele ratios.

All in all, our study confirms ddPCR as a feasible method for dd-cfDNA analysis in plasma from KTx patients, although we recommend prior genotyping of the donor and preamplification of cfDNA. In contrast to more complex and sophisticated next generation sequencing-based assays, it could be performed in any laboratory equipped with a ddPCR machine at a lower cost and results should be available within 2 days, with at most one additional day for one-time genotyping along with the first sample from each patient.

## Data Availability Statement

The raw data supporting the conclusions of this article will be made available by the authors, without undue reservation.

## Ethics Statement

The studies involving human participants were reviewed and approved by Slovenian National Medical Ethics Committee. The patients/participants provided their written informed consent to participate in this study.

## Author Contributions

MS and MR conceived the study. SV designed and performed SNP selection. MA recruited KTx patients for blood sample collection. MS organized sample storage, processing, and distribution. BJ and MŠ designed and performed the experiments, data analysis, and data interpretation. DD and PK supervised the work and provided critical insights. MŠ drafted the manuscript with the assistance of BJ. All authors revised and approved the final version of the manuscript.

## Funding

The presented work was financially supported by the Ministry of Education, Science and Sport of the Republic of Slovenia (operation Raziskovalci-2.0-NIB-529023, contract number C3330-17-529023) and the Slovenian Research Agency (research programmes P4-0165 and P4-0407).

## Conflict of Interest

The authors declare that the research was conducted in the absence of any commercial or financial relationships that could be construed as a potential conflict of interest.

## Publisher's Note

All claims expressed in this article are solely those of the authors and do not necessarily represent those of their affiliated organizations, or those of the publisher, the editors and the reviewers. Any product that may be evaluated in this article, or claim that may be made by its manufacturer, is not guaranteed or endorsed by the publisher.

## Footnotes

^1^ ftp://ftp.1000genomes.ebi.ac.uk/vol1/ftp/release/20130502/

## Abbreviations

cfDNA: cell-free deoxyribonucleic acid
CNA: QIAamp Circulating Nucleic Acid Kit
CV: coefficient of variation
dd-cfDNA: donor-derived cell-free deoxyribonucleic acid
ddPCR: droplet digital polymerase chain reaction
FPS: false positive signal
gDNA: genomic DNA
KTx: kidney transplant
ME: QIAamp MinElute ccfDNA Kit
MM: MagMAX Cell-Free DNA Isolation Kit
qPCR: real-time polymerase chain reaction
SNP: single nucleotide polymorphism.
