# Supplementary material for: Feasibility of Droplet Digital PCR Analysis of Plasma Cell-Free DNA From Kidney Transplant Patients
Source: Front Med (Lausanne). 2021 Oct 8;8:748668. doi: 10.3389/fmed.2021.748668 (PMC8531215; doi:10.3389/fmed.2021.748668)
Supplement: Supplementary file 1 [file Data_Sheet_1.PDF]

frontiers

Supplementary Material

1 Supplementary Figures

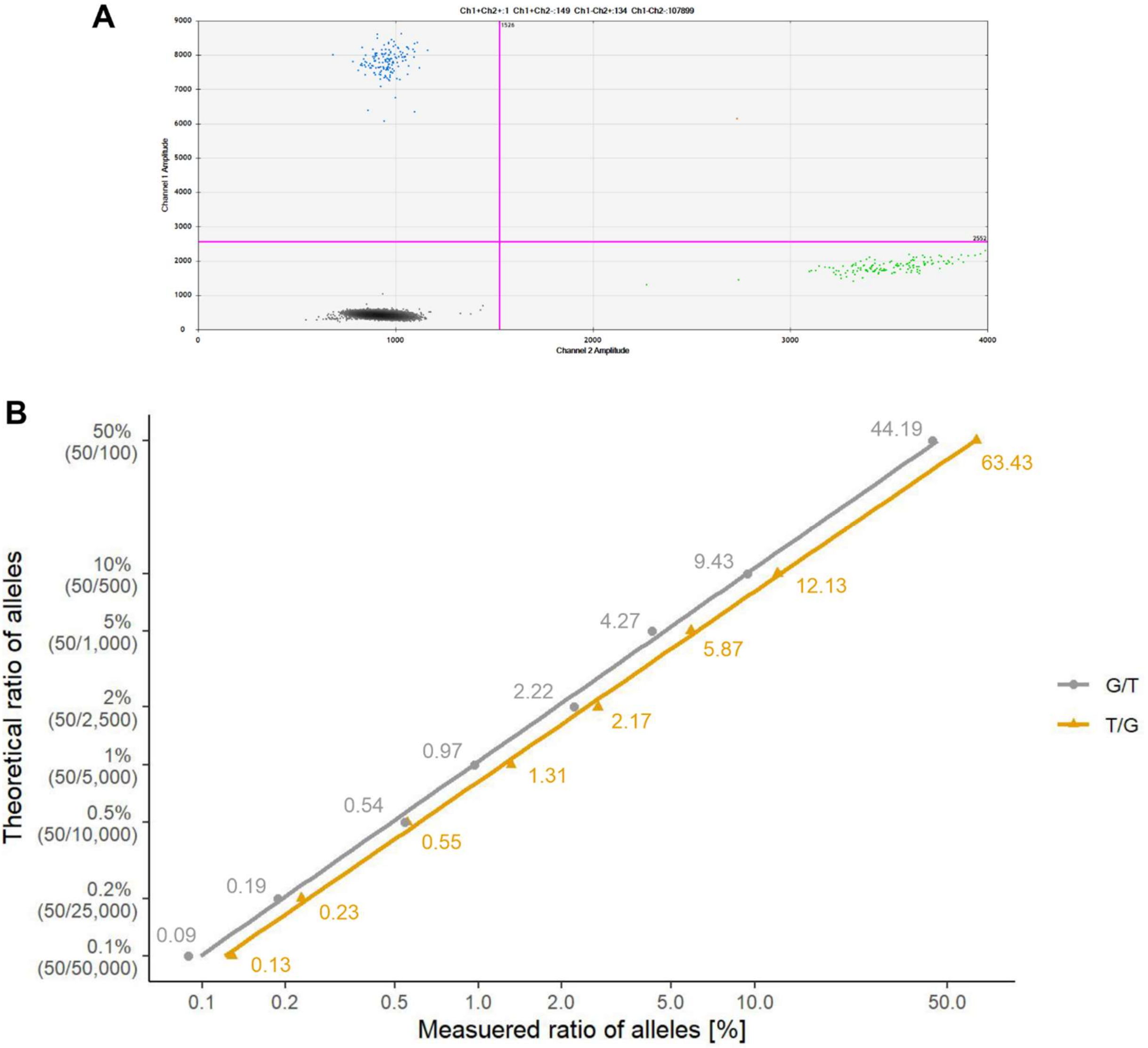

Supplementary Figure 1. Performance of the assay targeting rs1707473 in ddPCR. **(A)** 2D amplitude scatterplot shows signal for FAM (blue) and VIC (green) labeled probes, in a QX200 ddPCR reaction with 3-60 target allele copies per reaction. **(B)** Assay linearity at 0.1-50% allele1/allele2 ratio. The values next to the trend line show the actual measured ratios, while the ordinate represents the targeted (theoretically expected) values and the targeted copy numbers for each of the alleles.

1.1 Supplementary Tables

Supplementary Table 1. TaqMan genotyping SNP assays.

Target SNP	Forward primer sequence	Reverse primer sequence	VIC probe sequence	FAM probe sequence	Amplicon length [bp]
rs7431017	GCCTTAATAGCTTTAATTGCAGGATACAG	GTGACAGCTTATAGACAGGCAATGA	ACAGCTGCT**A**CTCCCT	CAGCTGCT**C**CTCCCT	89
rs1707473	AAGGGAAAAGTAAAATTCAAACAGCAAAGA	CCCCCAACTTTTTAAAGTCAAGTTTTACTC	TCCCCGCC**C**TGTGTGA	TCCCCGCC**A**TGTGTGA	93
rs2691527	TTGAGCCAGCCAAGACTATTCC	CCTCCTCCTCCAAAAGCAGAA	TTGCATGACTGA**G**TCACAG	TGCATGACTGA**A**TCACAG	70
rs7687645	CAGCAATGTACTTCTTCTCCATTCTCT	GGCCATTTCTCAGAATTATTTCACATTGA	CCTTCAGCT**T**CATCACC	CTTCAGCT**C**CATCACC	95
rs1420530	GGCCTCGACCATCCTCTCT	GTGGAGTTGTGAAAAGGCAGAAAAT	CTCAGAAAG**G**AGACCC	TCAGAAAG**A**AGACCC	67
rs9289628	CCTATAATTTTAGTGCCTGCAACCC	CCCCTTTCTTTGTCCTCCTTTACC	CAAATCAGTTGATC**G**TCCCAG	AATCAGTTGATC**A**TCCCAG	85
rs6070149	GTGCCAGACACAGGAGAGTAG	CCCAGGGCAATAATAGGAGAATCA	CCCTCAGA**G**ACAGGAC	CCCTCAGA**A**ACAGGAC	75

Supplementary Table 2. Results of ddPCR quantification of artificial 1% gBlock allele mixtures in evaluation of assay targeting rs1707473. Each mixture of alleles was tested in 6 parallel reactions and mean, SD and CV of measured copies per reaction (cp/20 ul) were calculated.

**Allele mixture** (allele 1/allele 2)	**Mean detected cp/20 µL reaction** (allele 1/allele 2)	**SD** (allele 1/allele 2)	**CV [%]** (allele 1/allele 2)
5/500	6.4/580.1	2.0/13.5	32.2/2.3
10/1000	10.2/1116.5	2.8/69.4	27.5/6.2
20/2000	20.3/2039.7	3.4/64.6	16.5/3.2
